# Impaired renal function impacts negatively on vascular stiffness in patients with coronary artery disease

**DOI:** 10.1186/1471-2369-14-173

**Published:** 2013-08-13

**Authors:** Sabrina H Rossi, Emily P McQuarrie, William H Miller, Ruth M Mackenzie, Jane A Dymott, María U Moreno, Chiara Taurino, Ashley M Miller, Ulf Neisius, Geoffrey A Berg, Zivile Valuckiene, Jonathan A Hannay, Anna F Dominiczak, Christian Delles

**Affiliations:** 1 Institute of Cardiovascular and Medical Sciences, BHF Glasgow Cardiovascular Research Centre, University of Glasgow, 126 University Place, G12 8TA, Glasgow, Scotland, UK; 2 Division of Cardiovascular Sciences, Center for Applied Medical Research, University of Navarra, Navarra, Spain; 3 Golden Jubilee National Hospital, Clydebank, UK; 4 Gartnavel General Hospital, Glasgow, UK

**Keywords:** Coronary artery disease, Chronic kidney disease, Vascular stiffness

## Abstract

**Background:**

Chronic kidney disease (CKD) and coronary artery disease (CAD) are independently associated with increased vascular stiffness. We examined whether renal function contributes to vascular stiffness independently of CAD status.

**Methods:**

We studied 160 patients with CAD and 169 subjects without CAD. The 4-variable MDRD formula was used to estimate glomerular filtration rate (eGFR); impaired renal function was defined as eGFR <60 mL/min. Carotid-femoral pulse wave velocity (PWV) was measured with the SphygmoCor® device. Circulating biomarkers were assessed in plasma using xMAP® multiplexing technology.

**Results:**

Patients with CAD and impaired renal function had greater PWV compared to those with CAD and normal renal function (10.2 [9.1;11.2] *vs* 7.3 [6.9;7.7] m/s; *P* < 0.001). In all patients, PWV was a function of eGFR (β = −0.293; *P* < 0.001) even after adjustment for age, sex, systolic blood pressure, body mass index and presence or absence of CAD. Patients with CAD and impaired renal function had higher levels of adhesion and inflammatory molecules including E-selectin and osteopontin (all *P* < 0.05) compared to those with CAD alone, but had similar levels of markers of oxidative stress.

**Conclusions:**

Renal function is a determinant of vascular stiffness even in patients with severe atherosclerotic disease. This was paralleled by differences in markers of cell adhesion and inflammation. Increased vascular stiffness may therefore be linked to inflammatory remodeling of the vasculature in people with impaired renal function, irrespective of concomitant atherosclerotic disease.

## Background

In patients with chronic kidney disease (CKD) cardiovascular risk is at least in part mediated by vascular stiffening. Vascular stiffness increases with progression of CKD [1] and is associated with cardiovascular mortality not only in patients with end stage renal disease (ESRD) [2] but also across all stages of CKD [3]. Vascular stiffness has also been found to be a predictor of decline in renal function in patients with CKD [4]. In patients with coronary artery disease (CAD) and normal renal function aortic stiffness is an independent predictor of major cardiac events [5]. Few studies, however, have examined the contribution of renal impairment to vascular stiffness in patients with established atherosclerotic disease. Covic *et al.*[6] demonstrated that aortic pulse wave velocity predicts the extent and severity of coronary artery disease in patients with CKD. We have previously demonstrated using cardiac magnetic resonance imaging (MRI) that aortic compliance is equally impaired in patients with CAD and in patients with ESRD [7]. More recently, Ilyas *et al*. [8] demonstrated in patients with various degrees of CAD that mild renal impairment is associated with increased arterial stiffness.

Inflammation and oxidative stress are key mechanisms in the development of vascular damage in atherosclerosis [9]. The increased vascular stiffness in patients with renal impairment, however, has also been attributed to arterial calcification, inflammation and oxidative stress [10,11]. It is unclear if renal impairment leads to acceleration of these mechanisms in patients with atherosclerotic disease. We therefore assessed carotid-femoral pulse wave velocity (PWV) as a direct marker of vascular stiffness in patients with and without CAD and studied whether mild to moderate renal impairment further increases arterial stiffness and if differences in PWV are paralleled by differences in markers of inflammation, cell adhesion and oxidative stress.

## Methods

### Participants

We included 160 patients with severe triple vessel CAD who underwent elective coronary artery bypass graft surgery at the Western Infirmary Glasgow between 2004 and 2008 [7,12-14]. Thirty nine patients with CKD stages 2 to 4 who attend renal clinics at the Western Infirmary Glasgow [15] and 130 healthy control subjects who were recruited from a local sports centre, from a surgical ward in Gartnavel General Hospital Glasgow or were Glasgow University employees, also participated in this study. Hence, a total of 329 subjects, 160 with CAD and 169 without CAD, were studied (Table 1). As a result of rigorous quality checks not all of the parameters below are available in all study participants.

**Table 1:** Characteristics of the study cohort

	**CAD**		**No CAD**	
	**n = 160**		**n = 169**	
	**eGFR ≥60 mL/min**	**eGFR <60 mL/min**	**eGFR ≥60 mL/min**	**eGFR <60 mL/min**
	**n = 105**	**n = 55**	**n = 119**	**n = 50**
Age (years)	62.2 [60.3;64.1]	69.4 [67.5;71.4]	57.0 [54.8;59.0]	60.2 [56.6;63.8]
Sex (% male)	90	49	55	22
Body mass index (kg/m^2^)	28.9 [27.9;29.9]	29.7 [28.5;30.9]	26.0 [25.4;26.7]	28.9 [27.1;30.7]
Active smoking (%)	18	10	10	14
Diabetes (%)	21	35	0	13
Systolic blood pressure (mmHg)	138 [134;143]	146 [139;154]	136 [133;139]	143 [138;149]
Diastolic blood pressure (mmHg)	77 [75;79]	78 [74;82]	80 [78;81]	80 [77;84]
Total cholesterol (mmol/L)	4.0 [3.8;4.2]	3.8 [3.5;4.0]	5.6 [5.4;5.9]	5.0 [4.2;5.8]
LDL cholesterol (mmol/L)	2.0 [1.9;2.2]	1.8 [.16;2.0]	3.4 [3.2;3.6]	2.8 [2.2;3.4]
HDL cholesterol (mmol/L)	1.1 [1.0;1.2]	1.2 [1.1;1.3]	1.5 [1.4;1.6]	1.5 [1.2;1.9]
Serum calcium (mmol/L)	2.29 [2.27;2.31]	2.37 [2.30;2.44]	2.35 [2.29;2.41];	2.39 [2.35;2.42]
Serum phosphate (mmol/L)	1.10 [1.03;1.16]	1.16 [1.05;1.27]	1.06 [0.88;1.24]	1.19 [1.11;1.27]
eGFR (mL/min)	76 [73;78]	47 [43;50]	82 [80;85]	32 [28;36]

*CAD* coronary artery disease, *eGFR* estimated glomerular filtration rate, *LDL* low density lipoprotein, *HDL* high density lipoprotein. Data are given as mean [95% confidence interval] or percentages as appropriate.

Demographic data were collected and a blood sample was taken to assess lipid profile, CRP, serum creatinine, calcium and phosphate using standard biochemical methods [7]. A lithium-heparinate sample was kept on ice for a maximum of 1 hour prior to centrifugation. Plasma was stored for biomarker analysis at −80°C. eGFR was determined using the 4-variable MDRD formula, calibrated to isotope dilution mass spectrometry reference. Impaired renal function was defined as eGFR <60 mL/min.

The study adheres to the principles of the Declaration of Helsinki and was approved by the West of Scotland Research Ethics Committee (Reference Number 06/S0703/110). All participants gave written informed consent.

### Pulse wave velocity and analysis

Carotid-femoral PWV was assessed using the SphygmoCor® device (AtCor Medical Ltd., Sydney, Australia) as previously described [16]. A measuring tape was used to assess the distance between the carotid and femoral artery recording sites. PWV was calculated automatically by dividing this distance by the time interval between the rapid upstroke in the pulse wave at the carotid and femoral arteries using the peak of the R-wave on electrocardiography as a reference point.

### Circulating markers of oxidative stress

Oxidised (GSSG) and reduced glutathione levels (GSH) were determined in whole blood using a photometric method (OXIS International Inc., Foster City, California, USA) as previously described [17]. Oxidised low-density lipoprotein (LDL) cholesterol (oxLDL) was determined using a competitive enzyme-linked immunosorbent assay (Mercodia AB, Uppsala, Sweden).

### Circulating biomarkers of inflammation and cell adhesion

Circulating biomarkers were assessed in plasma using three WideScreen multiplex assays (Merck, Nottingham, UK) on a Bio-Plex 100 xMAP platform (Bio-Rad, Hemel Hempstead, UK). WideScreen Human CVD Panel 2 allowed multiplex analysis of interleukin-6 (IL-6), macrophage inflammatory protein-1 alpha (MIP-1α), interleukin-8 (IL-8), macrophage inflammatory protein-1 beta (MIP-1β), monocyte chemoattractant protein 1 (MCP-1) and tumour necrosis factor alpha (TNF-α). WideScreen Human CVD Panel 3 was used to determine levels of E-selectin, P-selectin, intercellular adhesion molecule-1 (ICAM-1), Leptin, Osteopontin and soluble receptor of advanced glycation end-products (sRAGE). WideScreen Human CVD Panel 6 allowed determination of levels of Adiponectin, extracellular newly identified receptor for advanced glycation end-products binding protein (ENRAGE), plasminogen activator inhibitor-1 (PAI-1), Cystatin C and vascular cell adhesion molecule-1 (VCAM-1). Plasma samples were removed from −80°C storage, carefully thawed on ice, and prepared (diluted as required) according to the manufacturer’s instructions. Analyte levels were recorded from 100 microbeads per area of interest per sample, and each sample was analysed in duplicate using the Bio-Plex system, as *per* the manufacturer’s protocols. Data was processed and analysed using xPonent® Software (version 3.1.871; Merck, Nottingham, UK). Coefficients of variation were consistent with the manufacturer’s published data. Here we only report on biomarkers that passed the quality checks and were detected in plasma of our patients and control subjects; data for Cystatin C, interleukin-6 and MIP-1a did not pass the quality control or were out of the range of the standard curve.

### Mononuclear and whole cell superoxide production

Mononuclear cells were extracted from whole blood as previously described and diluted to 5×10^6^ cells/mL [18]. Superoxide production was measured in triplicate by electron paramagnetic resonance (EPR) spectroscopy (Bruker BioSpin e-scan R, Bruker Corporation, Rheinstetten, Germany) using the spin probe 1-Hydroxy-3-carboxy- 2,2,5,5-tetramethylpyrrolidine (CPH; Noxygen, Elzach, Germany) [19]. Maximum superoxide production was assessed after stimulation with phorbol 12-myristate 13-acetate (PMA, 3.2 μM; Sigma-Aldrich, Dorset, UK) and fold changes compared to basal production have been calculated. For assessment of whole blood superoxide generation blood was collected in lithium heparinate containing tubes, kept on ice and processed within half an hour. EPR measurements were performed after adding the spin probe CPH to a final concentration of 500 μmol/L [12]. Instrument settings were: microwave power, 22 mW; centre field, 3375 G; modulation amplitude, 2.27 G; sweep time, 5.24 s; sweep width, 60 G; 10 scans. Superoxide levels were recorded once a minute for 10 min and the rate of superoxide anion production was calculated as counts per minute. For mononuclear cells this has been standardised to a rate per 10^6^ cells per minute whereas for whole blood arbitrary units (AU) are presented.

### Statistical analysis

Statistical analysis was conducted using SPSS statistical package. Data are presented as mean [95% confidence interval] or median [interquartile range] as appropriate. Two sample Student’s t-test or Mann–Whitney U-test were used to compare data between groups of patients. Correlation was assessed by calculating Pearson’s (*r*) and Spearman’s (ρ) correlation coefficients as appropriate. Linear regression analysis was performed to show determinants of PWV using both a model where all variables were forced in and then a stepwise model (P_in_ < 0.05 and P_out_ > 0.10). For interaction analysis between eGFR and CAD status we centered eGFR around the mean of eGFR and calculated an interaction term by multiplying centered eGFR with CAD status (coded as 1 = “no CAD”, 2 = “CAD”); these terms were then used in linear regression analysis. A *P*-value of 0.05 (two sided) was considered significant.

## Results

### Study cohort

Clinical and demographic characteristics of study participants are presented in Table 1. As expected, patients with CAD and/or eGFR < 60 mL/min were older than control subjects, and due to lipid-lowering therapy, total and LDL cholesterol levels were lowest in patients with CAD. Blood pressure was similar across the study groups.

### Pulse wave velocity

**Figure 1:**
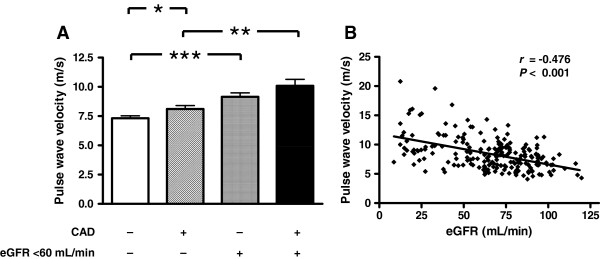
**Renal function and pulse wave velocity in the whole study cohort. A** Pulse wave velocity in participants with (+) or without (−) coronary artery disease (CAD) and with (+) or without (−) impaired renal function (estimated glomerular filtration rate [eGFR] <60 mL/min). * *P* < 0.05, ** *P* < 0.01; *** *P* < 0.001. **B** Scatterplot of pulse wave velocity *vs* eGFR calculated by the MDRD formula.

PWV was greater in patients with eGFR < 60 mL/min compared to those with eGFR ≥60 mL/min, both in patients without CAD (10.2 [9.1;11.2] *vs* 7.3 [6.9;7.7] m/s; *P* < 0.001) and with CAD (10.1 [8.9;11.2] *vs* 8.1 [7.5;8.7] m/s; *P* = 0.001). In those with an eGFR <60 mL/min, there was no difference in PWV between patients with and without CAD (*P* = 0.935). In contrast, PWV was greater in the CAD group when only patients with normal renal function were selected (*P* = 0.029) (Figure 1A).

Assessing eGFR as a continuous variable, a close correlation was found between PWV and eGFR (*r* = −0.476; *P* < 0.001) in the whole study cohort (Figure 1B). Linear regression analysis demonstrated that eGFR (β = −0.293; *P* < 0.001) contributed to PWV independently of age (β = 0.453; *P* < 0.001) and systolic blood pressure (β = 0.153; *P* = 0.009); whereas presence or absence of CAD (β = −0.015; *P* = 0.798), sex (β = 0.040; *P* = 0.523) and body mass index (β = 0.064; *P* = 0.294) did not contribute significantly. A model containing only age, systolic blood pressure and eGFR explained 42% of the variability of PWV.

Linear regression analysis to study interaction between CAD and eGFR confirmed the key role of eGFR as determinant of PWV. Centered eGFR (β = − 0.603, *P* = 0.002), but not CAD status (β-0.081; P = 0.191) and the interaction term (β = 0.137, *P* = 0.479) significantly contributed to the model explaining PWV.

### Oxidative stress

We explored if markers of oxidative stress differed between patients with CAD and those without CAD. In line with our previous observations [16,17] we found significantly increased levels of oxidative stress markers in patients with CAD compared with those without (Table 2). In contrast, no significant differences were found in patients with CAD when stratified into groups with normal or impaired renal function (Table 2). Oxidative stress therefore does not seem to explain the increased vascular stiffness associated with impaired renal function in patients with CAD.

**Table 2:** Markers of oxidative stress

	**CAD**			**No CAD**	***P*-value**
	**n = 100**			**n = 93**	**CAD vs no CAD**
	**eGFR ≥60 mL/min**	**eGFR <60 mL/min**	***P*-value**	**eGFR ≥60 mL/min**	
	**n = 67**	**n = 33**			
GSH (μmol/L)	992 [227]	894 [399]	0.067	1067 [270]	0.001
GSSG (μmol/L)	29.0 [63.5]	25.6 [71.1]	0.904	1.94 [24.5]	<0.001
GSH/GSSG ratio	32.9 [569]	32.6 [513]	0.636	177 [709]	0.001
OxLDL (AU)	59.5 [35.6]	50.2 [33.6]	0.111	54.8 [24.5]	0.847
OxLDL/LDL ratio (AU/mmol/L)	28.5 [16.9]	28.4 [10.9]	0.612	17.6 [5.7]	<0.001
Whole blood superoxide release (nmol/min/10^6^ celles)	1.6 [2.2]	1.3 [1.0]	0.143	1.2 [0.6]	0.012
Basal mononuclear cell superoxide release (nmol/min/10^6^ cells)	2.5 [2.0]	2.2 [1.8]	0.504	1.8 [1.0]	0.005
Maximum (PMA) mononuclear cell superoxide release (nmol/min/10^6^ cells)	28.1 [21.2]	30.5 [23.4]	1.000	14.7 [9.7]	<0.001
Fold change in mononuclear cell superoxide release	12.9 [7.1]	15.4 [9.2]	0.391	8.5 [5.4]	<0.001

*CAD* coronary artery disease, *GSH* reduced glutathione, *GSSG* oxidised glutathione, *OxLDL* oxidised low density lipoprotein cholesterol, *PMA* phorbol-12-myristate-13-acetate. Data are given as median [interquartile range]. *P*-values are derive from Mann–Whitney U-tests.

### Phosphate and circulating biomarkers of inflammation and cell adhesion

**Figure 2:**
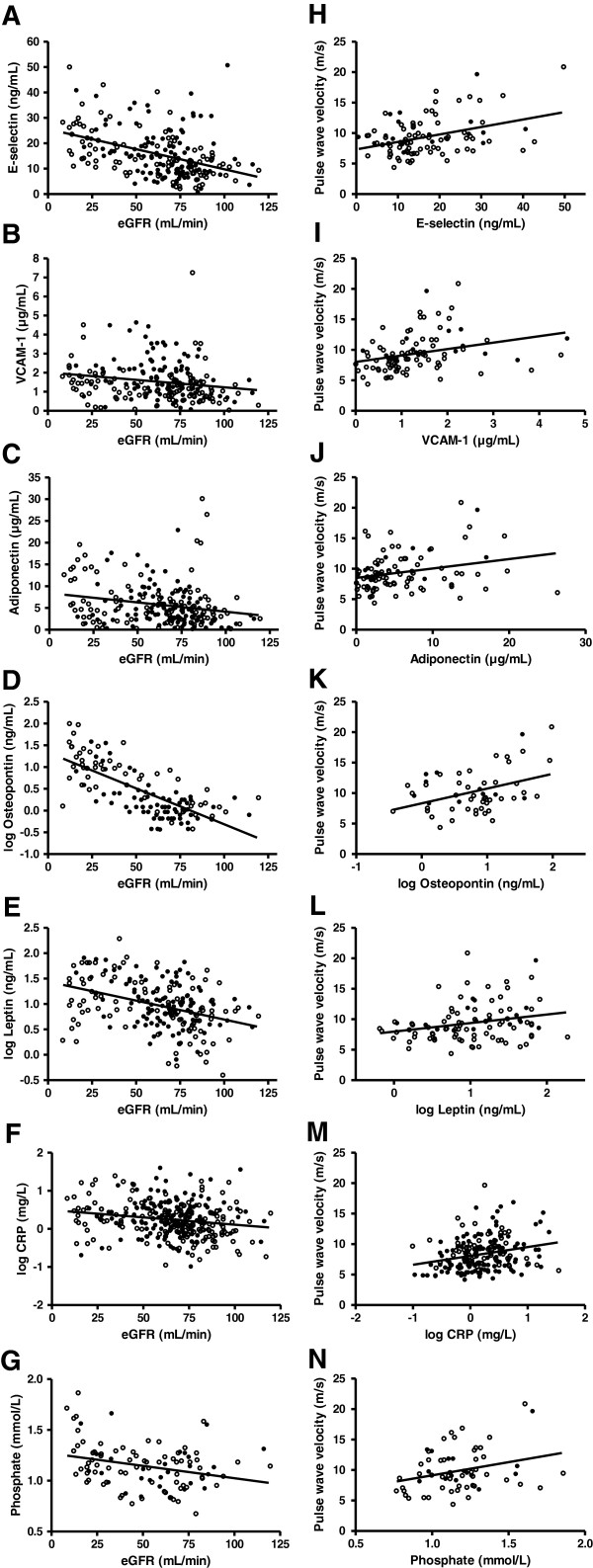
**Correlations between renal function, vascular stiffness and circulating biomarkers of inflammation and cell adhesion.** Panels **A** to **G** (left) show scatterplots of E-selectin, vascular cell adhesion molecule-1 (VCAM-1), adiponectin, osteopontin, leptin, C-reactive protein (CRP) and phosphate against estimated glomerular filtration rate (eGFR). Panels **H** to **N** (right) show scatterplots of pulse wave velocity (PWV) against these circulating markers. Data for osteopontin, leptin and CRP have been log transformed for these plots. Open symbols: no coronary artery disease; closed symbols: coronary artery disease. Spearman correlation coefficients and associated *P*-values are displayed in Table 4.

We then studied circulating biomarkers of inflammation and cell adhesion in control subjects and in patients with CAD of which both were stratified into groups with normal or impaired renal function. As expected, a number of markers were different between those with and without impaired renal function in the control group (Table 3). In a next step we analysed correlations between these markers and PWV and eGFR in the whole cohort (Table 4). We found that levels of E-selectin, VCAM-1, adiponectin, osteopontin and leptin were consistently different between groups with and without impaired renal function and were correlated with both eGFR and PWV. Scatterplots for these markers illustrating potential correlations that could provide a link between renal function and vascular stiffness are shown in Figure 2.

**Table 3:** Circulating biomarkers

	**CAD**			**No CAD**		
	**n = 109**			**n = 86**		
	**eGFR ≥60 mL/min**	**eGFR <60 mL/min**	***P*-value**	**eGFR ≥60 mL/min**	**eGFR <60 mL/min**	***P*-value**
	**n = 71**	**n = 38**		**n = 40**	**n = 46**	
E-selectin (ng/mL)	10.5 [10.4]	17.6 [12.6]	<0.001	10.8 [6.6]	19.2 [14.2]	<0.001
P-selectin (ng/mL)	90.4 [39.0]	106.1 [57.9]	0.015	85.6 [45.9]	113.0 [45.9]	0.001
ICAM-1 (ng/mL)	27.6 [11.4]	34.5 [21.9]	0.002	23.0 [12.6]	33.1 [15.6]	<0.001
VCAM-1 (μg/mL)	1.37 [1.26]	2.03 [1.36]	0.006	0.96 [0.75]	1.42 [0.95]	0.008
sRAGE (ng/mL)	3.8 [2.5]	4.8 [4.7]	0.110	3.8 [3.0]	6.7 [5.9]	<0.001
ENRAGE (ng/mL)	73.2 [81.0]	65.3 [149.9]	0.794	53.9 [56.9]	38.3 [32.2]	0.059
IL-8 (pg/mL)	4.0 [3.7]	3.8 [4.1]	0.218	3.2 [3.0]	5.1 [4.4]	0.005
MCP-1 (pg/mL)	44.6 [31.8]	50.8 [48.1]	0.488	54.9 [40.2]	70.3 [53.7]	0.009
MIP-1β (pg/mL)	66.2 [50.4]	73.5 [56.5]	0.258	66.4 [48.2]	95.0 [91.1]	0.029
CRP (mg/L)	2.0 [2.2]	2.4 [2.9]	0.138	1.3 [1.9]	2.9 [4.8]	<0.001
TNF-α (pg/mL)	0.73 [1.14]	0.96 [1.63]	0.182	0.65 [0.62]	1.73 [1.29]	<0.001
PAI-1(ng/mL)	219.0 [198.6]	152.5 [265.8]	0.329	175.8 [221.0]	153.8 [101.8]	0.044
Adiponectin (μg/mL)	4.1 [3.9]	5.6 [6.9]	0.020	3.5 [4.6]	5.9 [9.3]	0.047
Osteopontin(ng/mL)	0.5 [1.0]	1.8 [4.0]	<0.001	0.0 [0.9]	9.4 [17.9]	<0.001
Leptin (ng/mL)	9.1 [8.0]	15.0 [27.3]	0.011	6.4 [9.0]	16.3 [28.8]	<0.001

*CAD* coronary artery disease, *eGFR* estimated glomerular filtration rate, *ICAM-1* intercellular adhesion molecule-1, *VCAM-1* vascular cell adhesion molecule-1, *sRAGE* soluble receptor of advanced glycation end-products, *ENRAGE* extracellular newly identified receptor for advanced glycation end-products binding protein, *MCP-1* monocyte chemoattractant protein 1, *MIP-1β* macrophage inflammatory protein-1 beta, *CRP* C-reactive protein, *TNF-α*, tumour necrosis factor alpha, *PAI-1* plasminogen activator inhibitor-1. Data are given as median [interquartile range]. *P*-values refer to comparisons between the eGFR ≥60 mL/min and eGFR <60 mL/min groups.

**Table 4:** Correlations between biomarkers and PWV and eGFR

	**eGFR**		**PWV**	
	**ρ**	***P*-value**	**ρ**	***P*-value**
E-selectin	−0.441	<0.001	0.281	0.006
P-selectin	−0.272	<0.001	0.125	0.228
ICAM-1	−0.541	<0.001	0.193	0.061
VCAM-1	−0.264	<0.001	0.417	<0.001
sRAGE	−0.369	<0.001	0.052	0.615
ENRAGE	0.086	0.215	0.143	0.167
IL-8	−0.208	0.002	0.106	0.305
MCP-1	−0.203	0.003	0.069	0.505
MIP-1β	−0.130	0.061	0.185	0.073
C-reactive protein	−0.214	<0.001	0.272	<0.001
TNF-α	−0.323	<0.001	0.218	0.034
PAI-1	0.157	0.023	0.072	0.489
Adiponectin	−0.239	<0.001	.0245	0.017
Osteopontin	−0.602	<0.001	0.377	<0.001
Calcium	−0.312	0.001	−0.126	0.317
Phosphate	0.977	<0.001	0.261	0.037
Calcium × Phosphate	−0.329	0.001	0.207	0.101
Leptin	−0.384	<0.001	0.268	0.009

*eGFR* estimated glomerular filtration rate, *PWV* pulse wave velocity, *ICAM-1* intercellular adhesion molecule-1, *VCAM-1* vascular cell adhesion molecule-1, *sRAGE* soluble receptor of advanced glycation end-products, *ENRAGE* extracellular newly identified receptor for advanced glycation end-products binding protein, *MCP-1* monocyte chemoattractant protein 1, *MIP-1β* macrophage inflammatory protein-1 beta, *TNF-α* tumor necrosis factor alpha, *PAI-1* plasminogen activator inhibitor-1. Spearman correlation coefficients (ρ) and associated *P*-values are given.

When added to the multivariate model to study the association of the markers with PWV independently of eGFR, age, systolic blood pressure, sex, presence or absence of CAD and body mass index only E-selectin (β = 0.307, *P* < 0.001; also included: age [β = 0.617, *P* < 0.001]), osteopontin (β = 0.336, *P* < 0.001; also included: age [β = 0.538, *P* < 0.001], body mass index [β = 0.188, *P* = 0.021]) and adiponectin (β = 0.203, *P* = 0.017; also included: age [β = 0.502, *P* < 0.001], eGFR [β = − 0.186, *P* = 0.036] and systolic blood pressure [β = 0.215, *P* = 0.014]) were part of the final models. Of note, the contribution of osteopontin to PWV remained statistically significant even after additional adjustment for phosphate (osteopontin, β = 0.206, *P* = 0.049; also included: age [β = 0.638, *P* < 0.001] and systolic blood pressure [β = 0.205; *P* = 0.046]) or calcium (osteopontin, β = 0.287, *P* = 0.005; also included: age [β = 0.631, *P* < 0.001]). Phosphate, CRP, VCAM-1 and leptin were no significant determinants of PWV when adjusted for other factors.

## Discussion

In this study we demonstrate that arterial stiffness is greater in individuals with impaired renal function compared to those with eGFR ≥60 mL/min, even in the presence of advanced CAD. The greater vascular stiffness in the group with impaired renal function was paralleled by differences in markers of inflammation and cell adhesion but not by markers of oxidative stress. We found a close relationship between renal function and PWV, suggesting that renal impairment has an incremental negative effect on arterial stiffness in patients with established CAD. Indeed, impaired renal function was found to be a far stronger determinant of PWV than presence of CAD itself.

### Vascular stiffness

Increased vascular stiffness is an independent predictor of cardiovascular outcome [2,20,21]. Impaired renal function is associated with vascular stiffening, but the underlying mechanisms are incompletely understood [1,10,11,22,23]. From a recent study by Ilyas *et al*. [8] it appears that there is an additive contribution of impaired renal function to vascular stiffness even in patients with moderately severe CAD. In our present study we have gone one step further and studied patients with severe triple vessel CAD who require surgical revascularisation, compared them with patients without CAD and assessed a wide range of markers of oxidative stress, inflammation and cell adhesion.

We have previously reported reduced aortic compliance in patients with ESRD [7]. In the present study we not only found a linear relationship between renal excretory function and vascular stiffness in mild-to-moderate renal impairment, but also demonstrated that this association is independent of other cardiovascular risk factors including systolic blood pressure and age.

### Circulating biomarkers and oxidative stress

Inflammatory processes play an important role in the pathogenesis of vascular disease [9], and renal impairment has been identified as a chronic low grade inflammatory state [11]. The present study demonstrates that levels of E selectin and VCAM-1 are increased in patients with CAD and renal impairment relative to patients with CAD alone. In multivariate analysis only E-selectin was an independent predictor of PWV, and the final model only contained age and E-selectin but not eGFR or blood pressure. It therefore appears plausible, although not directly proven in this cross-sectional study, that effects of impaired renal function on vascular stiffness are at least in part mediated by increased levels of E-selectin. We have previously demonstrated a role of E-selectin in the early stages of pre-eclampsia, a condition that is also associated with renal damage and endothelial dysfunction [24]. Other studies [25,26] reported that adhesion molecules are independent predictors of cardiovascular events in patients with ESRD, although not all studies confirm these data [27]. Adhesion of inflammatory cells to the endothelium is an early step in the development of vascular disease and further induces local vascular inflammation [28].

We have also found independent associations of osteopontin and adiponectin with vascular stiffness. Osteopontin is an extracellular matrix protein that contributes to the development of atherosclerosis as it acts as a pro-inflammatory cytokine to induce macrophage adhesion and migration, it promotes vascular smooth muscle cell proliferation and may mediate vascular calcification [29]. Osteopontin mRNA is expressed in aortic atherosclerotic plaques in patients with CAD, but not in the arteries of healthy controls, and expression is proportional to severity of disease [30]. In a prospective study of patients with established CAD, osteopontin was an independent predictor of fatal and nonfatal cardiovascular events even after adjustment for traditional risk factors [31]. Lorenzen *et al.*[29] demonstrated that in patents with CKD, osteopontin levels increase linearly with progressive decline in GFR. Our study suggests that osteopontin levels are further increased in patients with CAD and concomitant renal impairment, and thus may contribute to the development of vascular disease in this group. It is important to note that the association between osteopontin and vascular stiffness in our study was independent of calcium or phosphate levels. The findings on adiponectin are more difficult to interpret. Adiponectin is an adipose tissue-derived protein that is generally thought to have vasoprotective properties. Lower levels of adiponectin have been found in a number of cardiovascular conditions including diabetes [32] and hypertension [33]. On the other hand, renal failure is associated with reduced clearance of adiponectin, and a recently proposed interaction between cystatin C and adiponectin could explain the paradoxical finding of high adiponectin levels in renal failure and the absence of a vasoprotective effect under these conditions [34].

Oxidative stress has been proposed as a unifying concept to explain the pathophysiology of cardiovascular disease in uraemic patients [11]. We have previously shown that vascular superoxide production is a significant determinant of vascular stiffness in patients without renal disease [16]. Levels of reactive oxygen species are increased in patients with CAD [12,17], but in our present study there was no further rise in oxidative stress to explain the increased vascular stiffness in those with concomitant renal impairment. However, increased expression of adhesion molecules may lead to increased numbers of inflammatory cells in the vessel wall and thereby promote functional and structural changes.

### Limitations

A limitation of our study is that renal impairment was assessed by estimating GFR from serum creatinine using the MDRD equation. eGFR is a measure of renal function which also integrates other important determinants of vascular stiffness, namely age and sex. Without exact measurement of GFR (*e.g.* by inulin clearance) we cannot precisely separate the effect of renal impairment from that of age on vascular stiffness. However, this issue has been raised in other studies as well and it is not necessarily a limitation [35]. In fact, eGFR captures risk related to a number of characteristics and is an excellent surrogate marker in clinical practice [35,36]. We acknowledge, however, that assessment of renal function at a single time point does not take variability of renal function in response to diet, fluid intake and medication into account.

Another limitation of this study is the cross-sectional design, which precludes inferences about the contribution of renal impairment on vascular stiffness and the effect on future cardiovascular risk, especially as vascular stiffening itself is a process progressing over a long period of time, where improvements of vascular stiffness are associated with improved outcome in patients with CAD [37]; rather we must limit ourselves to showing differences between patient groups. However, vascular stiffness is an established independent predictor of cardiovascular outcome in the general population [38,39], in patients with CAD [21] and in individuals on dialysis [2,40]. Recently, Ilyas *et al.*[8] suggested that assessment of arterial stiffness in patients with established CAD and mild renal impairment has some prospective value, as lower GFR and higher PWV predicted a shorter time to cardiovascular hospitalization and all cause mortality.

We also acknowledge that we did not control for drug therapy in this study. Therefore a number of potentially important factors, in particular lipid levels that are affected by statin therapy which is especially prevalent in patients with CAD, may not be adequately represented in our analyses.

Finally, residual confounding is a potential limitation of any study using multivariate analysis. We apreciate that other factors than renal function may have additional effects on vascular stuffness.

## Conclusions

In summary we have demonstrated that even in patients with advanced atherosclerotic disease, concomitant renal impairment is associated with a further increase in vascular stiffness. Our study highlights the pre-eminent role of renal function as a cardiovascular risk factor and justifies efforts to preserve renal function, especially in patients with established cardiovascular diseases. Due to the cross-sectional design, we were unable to demonstrate a direct causal relationship between the changes seen in circulating biomarkers and vascular stiffness. However, the pattern seen suggests that renal impairment may aggravate vascular disease by effects on cell adhesion and inflammation.

## Abbreviations

AU: Arbitrary units; CAD: Coronary artery disease; CKD: Chronic kidney disease; CPH: 1-Hydroxy-3-carboxy- 2,2,5,5-tetramethylpyrrolidine; CRP: C-reactive protein; eGFR: Estimated glomerular filtration rate; ENRAGE: Extracellular newly identified receptor for advanced glycation end-products binding protein; EPR: Electron paramagnetic resonance; ESRD: End-stage renal disease; GSH: Reduced glutathione; GSSG: Oxidised glutathione; IL-6: Interleukin-6; IL-8: Interleukin-8; LDL: Low-density lipoprotein; MCP-1: Monocyte chemoattractant protein 1; MIP-1α: Macrophage inflammatory protein-1 alpha; MIP-1β: Macrophage inflammatory protein-1 beta; oxLDL: Oxidised low-density lipoprotein; PAI-1: Plasminogen activator inhibitor-1; PMA: Phorbol 12-myristate 13-acetate; PWV: Pulse wave velocity; sRAGE: Soluble receptor of advanced glycation end-products; TNF-α: Tumour necrosis factor alpha; VCAM-1: Vascular cell adhesion molecule-1; VCAM-1: Vascular cell adhesion molecule-1.

## Competing interests

WHM received support towards travel and accommodation from Merck Chemicals Ltd. to present preliminary data of this study. There is no other conflict of interest.

## Authors’ contributions

Sabrina H Rossi acquired and analysed data; Emily P McQuarrie acquired data, recruited patients with CKD and drafted the manuscript; William H Miller acquired data on markers of inflammation and cell adhesion and drafted the manuscript; Ruth M Mackenzie acquired data on markers of inflammation and cell adhesion; Jane A Dymott acquired data and recruited patients with CAD; María U Moreno acquired data on whole blood and mononuclear cell superoxide production; Chiara Taurino acquired data on whole blood and mononuclear cell superoxide production and recruited patients with CAD; Ashley M Miller acquired data on markers of inflammation and cell adhesion; Ulf Neisius acquired data and recruited patients with CAD; Geoffrey A Berg acquired data and recruited patients with CAD; Zivile Valuckiene acquired data and recruited patients with CAD; Jonathan A Hannay acquired data and recruited control subjects; Anna F Dominiczak designed the research; Christian Delles designed the research, acquired and analysed the data, and drafted the manuscript. All authors have critically revised the manuscript and have given final approval of the version to be published.

## Pre-publication history

The pre-publication history for this paper can be accessed here:

http://www.biomedcentral.com/1471-2369/14/173/prepub
